# Photosensitizer-Functionalized Nanocomposites for Light-Activated Cancer Theranostics

**DOI:** 10.3390/ijms22136658

**Published:** 2021-06-22

**Authors:** Banendu Sunder Dash, Suprava Das, Jyh-Ping Chen

**Affiliations:** 1 Department of Chemical and Materials Engineering, Chang Gung University, Kwei-San, Taoyuan 33302, Taiwan; banendusunder@gmail.com (B.S.D.); supravadas0603@gmail.com (S.D.); 2 Craniofacial Research Center, Department of Plastic and Reconstructive Surgery, Chang Gung Memorial Hospital, Linkou, Kwei-San, Taoyuan 33305, Taiwan; 3 Research Center for Food and Cosmetic Safety, Research Center for Chinese Herbal Medicine, College of Human Ecology, Chang Gung University of Science and Technology, Taoyuan 33305, Taiwan; 4 Department of Materials Engineering, Ming Chi University of Technology, Tai-Shan, New Taipei City 24301, Taiwan

**Keywords:** photosensitizer, cancer therapy, nanocomposite, photodynamic therapy, photothermal therapy

## Abstract

Photosensitizers (PSs) have received significant attention recently in cancer treatment due to its theranostic capability for imaging and phototherapy. These PSs are highly responsive to light source of a suitable wavelength for image-guided cancer therapy from generated singlet oxygen and/or thermal heat. Various organic dye PSs show tremendous attenuation of tumor cells during cancer treatment. Among them, porphyrin and chlorophyll-based ultraviolet-visible (UV-Vis) dyes are employed for photodynamic therapy (PDT) by reactive oxygen species (ROS) and free radicals generated with 400–700 nm laser lights, which have poor tissue penetration depth. To enhance the efficacy of PDT, other light sources such as red light laser and X-ray have been suggested; nonetheless, it is still a challenging task to improve the light penetration depth for deep tumor treatment. To overcome this deficiency, near infrared (NIR) (700–900 nm) PSs, indocyanine green (ICG), and its derivatives like IR780, IR806 and IR820, have been introduced for imaging and phototherapy. These NIR PSs have been used in various cancer treatment modality by combining photothermal therapy (PTT) and/or PDT with chemotherapy or immunotherapy. In this review, we will focus on the use of different PSs showing photothermal/photodynamic response to UV-Vis or NIR-Vis light. The emphasis is a comprehensive review of recent smart design of PS-loaded nanocomposites for targeted delivery of PSs in light-activated combination cancer therapy.

## 1. Introduction

Photosensitizers (PSs) have been used as light-responsive drugs with their imaging capabilities, easy synthesis, tunable energy levels, and biocompatibility, which make them an innovative tool for therapeutic approaches in treatment of various cancers [1,2,3,4,5,6,7]. Among different methods for cancer therapy, photodynamic therapy (PDT) using a PS can take advantage of penetrated light into a targeted tissue for improving tumor control. The PDT comprises of three major components; PS, oxygen, and light source of appropriate wavelength [8], which when act together, generate a photochemical reaction. Depending on the part of the body being treated, the photosensitizing agent is either administered into the bloodstream through a vein or placed directly on the skin. By irradiation light on the targeted area, the excited PS emits energy in the form of heat and exhibits intersystem crossing (ISC), leading to the formation of reactive oxygen species (ROS) in triplet state [9].

The mechanism of killing cancer cells by activating PS with light has been revealed to be composed of three major mechanisms, apoptotic, autophagy, and necrotic cell death. The PSs for PDT could be classified into two categories, non-porphyrinoid based PSs (first generation PSs) and porphyrinoid based PSs (second generation PSs). The non-porphyrinoid PSs were limited by requiring the use of shorter wavelength (<630 nm) light source [10]. A complex mixtures of PSs may demand confinement of the treated patient in the dark to avoid skin photosensitization [11]. Photofrin is the most commonly used commercially available PS approved by the U.S. Food and Drug Administration (FDA), a hematoporphyrin derivative belonging to the porphyrin family. In 1841, Scherer introduced hematoporphyrin [12] and the observation of porphyrin fluorescence from treated tumors was published by Policard in 1924 [13]. The first generation compounds also have the demerits of suboptimal tumor selectivity, tumoricidal depth [14], and being a complex mixture of uncertain compounds [15]. The second generation PSs is purer in a chemical sense and offer high quantum yields for the triplet state generation and ROS formation. A group led by Hasan has done a significant number of studies relating to the combination of PDT with other forms of cancer treatment methods [16,17]. Though it is very hard to anticipate the efficacy of both types of PSs, the second generation PSs have still been found to be more reliable in complex biological environments.

For effective implementation, the uptake of PSs in cancer cells and their retention in cancer cells have been studied, including mechanism of tumor response and temperature sensitivity throughout the treatment period. Two of the main advantages of second generation PSs are the intense absorption in the red light wavelength and limited skin photosensitization. Graphene-based nanomaterials are good photothermal agents for photothermal therapy (PTT) [18]. However, for intracellular imaging, they need to be labeled with various fluorescein dyes like fluorescein isothiocyanate (FITC), quantum dots, and cyanine 5 (Cy 5) [19,20]. As PSs, near infrared (NIR) dyes not only could be used for fluorescence imaging but also could be a suitable tool for PDT/PTT. Various NIR organic dyes are endowed with photodynamic properties and show promising results in PDT-based cancer treatment [21,22,23]. Compared with ultraviolet-visible (UV-Vis) dyes for imaging and PDT, the ICG derivatives show deep tissue penetration depth, useful for imaging as well as for PTT/PDT with their good photothermal conversion upon NIR light activation [24,25,26,27]. This review provides up-to-date discussion about applications of different types of UV-Vis and NIR-activated PSs in a nanocomposite form for cancer therapy. The advantages and disadvantages of using these PSs for tumor destruction, which is directly related to their cancer treatment efficacy, are described. In addition, we aim to discuss the benefits of using PSs for PTT and/or PDT, while combining with other cancer therapeutic agents for combination cancer therapy.

## 2. Functions of Photosensitizers (PSs)

### 2.1. Imaging

**Figure 1:**
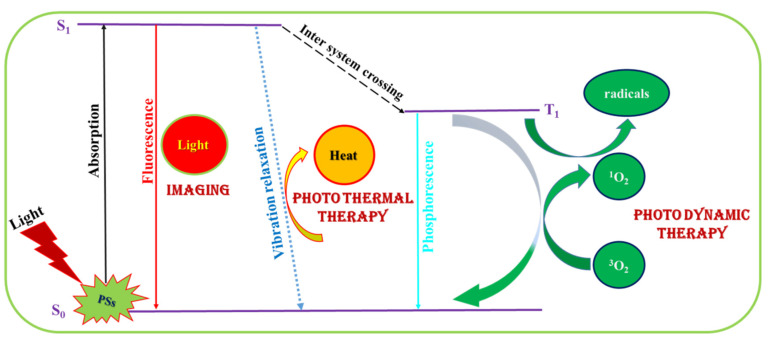
The Jablonski diagram showing the process of fluorescence imaging as well as photothermal therapy and photodynamic therapy when a photosensitizers (PS) is irradiated with light.

Through multimodal imaging techniques to determine the response during PDT, signals recorded during light irradiation can be easily depicted and monitored. A PS can thus be used both as an imaging agent in addition to a light-activated therapeutic agent, as shown in Figure 1. After delivery of a PS into the targeted tissue, it can act as an imaging moiety upon light exposure by emitting fluorescence signal. The PS 5-aminolevulinic acid (5-ALA) has been deployed as an intraoperative optical imaging agent for direct visualization of tumor tissue during fluorescence guided surgery [28]. The fluorescence emitted by 5-ALA is useful for diagnose of cancers in pre-malignancy and different malignancy stages [29]. As a PS, Chlorin e6 (Ce6) has the tendency to aggregate at room temperature. Hence, Ce6 was embedded within polyvinylpyrrolidone (PVP) for interfaced with a fluorescence endoscope system directly for photodynamic diagnosis of nasopharyngeal carcinoma [30]. The fluorescence properties of the NIR PS ICG were studied in aqueous solutions, from which concentration-dependent quenching effect was found to directly impact its fluorescence property [31]. By accumulation of an imaging-guided PS in tumor tissues, it can help to increase fluorescence intensity over time. Using NIR emission-centered PSs will give higher fluorescence quantum yield, rendering better performance as imaging agents.

### 2.2. Photodynamic Therapy (PDT)/Photothermal Therapy (PTT)

In molecular spectroscopy, a Jablonski diagram represents the energy diagram accompanied by a molecule’s electronic states and the transitions between them (Figure 1). The energy dissipation involves the movement of electrons upon excitation from the ground state to a higher energy state when UV-Vis or NIR light absorption occurs. In accordance with the transition between different spin states (denoted by transition between S_1_ to T_1_), intersystem crossing (ISC) of states are arranged vertically by energy, and grouped horizontally by spin multiplicity. Non-radiative vibration relaxation that leads to photothermal effects is shown by dotted arrow. The radiative transition is shown by straight solid arrow. The transition of triplet oxygen to singlet oxygen leads to formation of reactive oxygen species (ROS), leading to photodynamic effects. Radical formation from the excited state can also cause damage to cancer tissue for phototherapy in the NIR region. In order to avoid redundant interference of drugs in whole organism, the PDT is well pronounced for treatment of targeted tissue only. For PDT in cancer treatment, a light-sensitive PS was administered intravenously, orally, or topically, depending on the location of tumor, followed by accumulation of the PS at the desired targeted site. A light source of appropriate wavelength is applied to the targeted site to generate ROS for killing the cancer cells without affecting healthy tissues. The cell death triggered by PDT can occur through apoptosis and necrosis; the apoptotic pathway is referred to as programmed cell death, whereas necrotic pathway is designated as non-programmed cell death. An apoptotic pathway can shift into the necrotic pathway over decrease in the availability of caspases and intracellular adenosine triphosphate (ATP) concentration [32]. In addition, another cell mechanism called paraptosis can also lead to cell death with photo damage to the endoplasmic reticulum during PDT, by initiating impaired apoptotic pathways [33].

## 3. Photosensitizers (PSs) for Cancer Theranostics

**Figure 2:**
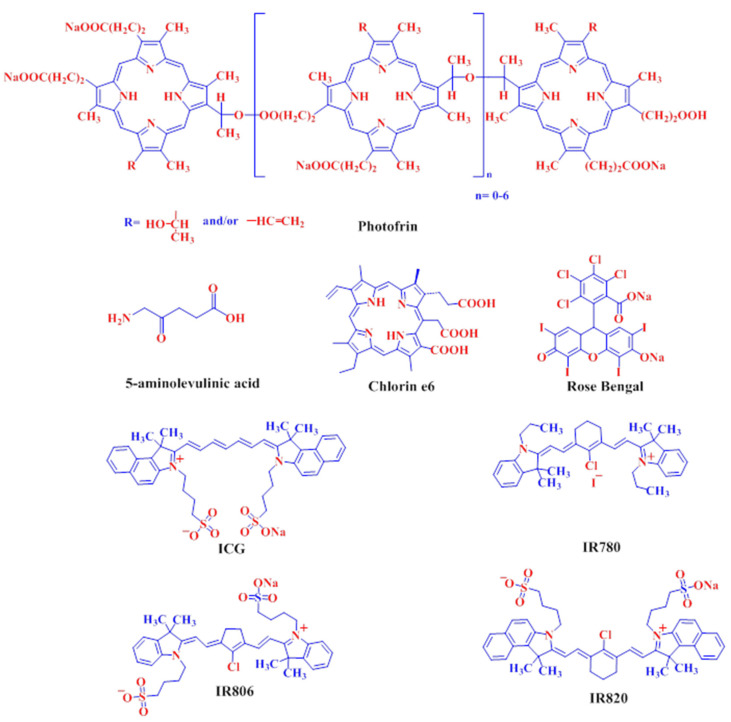
The chemical structure of ultraviolet-visible (UV-Vis) photosensitizers (top row) and near infrared (NIR) photosensitizers (bottom row).

In order to implement PDT for cancer therapy, various PSs have been developed. These PSs could be classified into two types. The lower wavelength based PSs (e.g., Photofrin, Chlorin e6, Rose Bengal and 5-aminolevulinic acid) (Figure 2) are efficiently activated by light sources in the UV-Vis range (400–700 nm), which show limited penetration into the tissue. On the other hand, the longer wavelength-based PS (e.g., ICG and IR780) (Figure 2) are mostly activated by light in the higher NIR wavelength range (700–900 nm), which show intense penetration ability with excellent anti-cancer efficacy. However, these PSs are still limited by factors like agglomeration and insolubility in aqueous solutions, leading to limited generation of ROS. To overcome these problems, new NIR PSs (e.g., IR806 and IR820), showing better aqueous suspension ability and better imaging properties, were developed recently for in vitro and in vivo use (Figure 2). From a comprehensive literature search, we find out more than 1000 research articles published within the last decade on UV-Vis PSs for cancer therapy, within which more than half are using Photofrin and 5-aminolevulinic acid. For NIR PSs, the number is more than 700, with ICG accounting for ~60% of them. For clinical trials, 5-aminolevulinic acid is used for basal cell carcinoma (phase I and 2); Photofrin is used for brain tumor (phase 1 and 2) and esophageal adenocarcinoma (phase 3); Rose Bengal is used for melanoma (phase 1 and 2); ICG is used for head and neck cancer (phase 2) and breast cancer (phase 2).

### 3.1. UV-Vis Photosensitizers

The UV-Vis PSs are dyes excited by blue, green, and red light within 400–700 nm. Herein, we discussed the advances in incorporating these PSs in a nanocomposite for treating different types of cancer in vitro and in vivo.

#### 3.1.1. Porfimer Sodium (Photofrin)

In the past, porfimer sodium was widely used as a PS for cancer treatment as Photofrin, which was approved by the U.S. FDA for treatment of specific kinds of cancer like esophagus and lung cancer. In 1995, Tsukagoshi combined porfimer sodium with laser as a new method for cancer therapy by selective accumulation of the PS, Photofrin II, in cancer tissues and further irradiation with light to cause tumor cell death [34]. Tumor selectivity of porfimer sodium is based on the following reasons, high affinity for lipoprotein, especially low-density lipoprotein (LDL); elevation of LDL receptor activity in cancer tissue; lack or incompleteness of lymphatic system in cancer tissue. By stimulation with 630 nm light, highly reactive excited singlet oxygen (^1^O_2_) was produced, which can kill tumor cells through mitochondrial apoptosis due to the release of cytochrome c triggering photodamage in the cytoplasm. Somers et al. carried out a comparative study using Photofrin-mediated PDT on squamous cell carcinoma (SqCCa) of the larynx [35]. In their experiments, 26 patients were taken with early stage of SqCCa, who have failed prior to surgery and/or radiation therapy. Outcomes of this study demonstrated potential treatment of early-stage laryngeal malignancies from multiple Photofrin administration and laser retreatment. The Photofrin mainly interacted with mitochondrial membranes, endoplasmic reticulum, and Golgi complexes of cancer cells. Under the irradiation process, singlet oxygen species and other ROS are produced, causing photo-oxidative damage to proteins and lipids of cancer cells that reside within a few nanometers of the PS. Cai et al. used Photofrin for PDT, in combination with glycated chitosan (GC) as an immunomodulatory agent for laser immunotherapy (LIT), in treating mice bearing EMT6 breast tumors and 4T1 metastatic breast tumors [36]. The long-term effects of LIT help to improve the survival rate of mice as well as minimizing the growth of the tumor.

In another study, Betsy et al. used modified Photofrin for breast cancer treatment. They modified the PS with picolylamine groups and its zinc complex compound to kill MDA-MB-231 cancer cells, finding that picoly Photofrin can enhance singlet oxygen production [37]. Choi and co-workers studied the effectiveness of generated ROS in cancer treatment with HeLa cells using Photofrin for PDT, together with a chemotherapeutic drug (carboplatin) [38]. Combination of Photofrin with carboplatin results in better cancer treatment outcomes than using Photofrin alone. Zhu et al. studied sinoporphyrin sodium and Photofrin-mediated PDT against HCT116 cells [39]. They observed that sinoporphyrin sodium-based PDT shows better anti-tumor efficiency both in vitro and in vivo as compared to Photofrin-based PDT, suggesting that sinoporphyrin sodium can be also used as an effective UV-Vis PS. Kano and co-worker introduced a new approach in which Photofrin was complexed with tumor-localizing polyethylene glycol-grafted poly(l-lysine) (PLL-g-PEG) for accumulating the PS at the tumor [40]. During the complexation process, Photofrin could be strongly bound to PLL-g-PEG due to both ionic and hydrophobic interactions, and the nanocomposite comprising Photofrin and PLL-g-PEG provides better anti-cancer performance tested with 4T1 and CT26 cancer cells over pristine Photofrin. Crescenzi et al. carried out comparative study between monotherapy (PDT or chemotherapy) and combination therapy (PDT plus chemotherapy) using Photofrin as the PS, as well as cisplatin and gemcitabine as cytotoxic drugs [41]. Using lung cancer cell line (H1299), they concluded that combination chemo-photodynamic therapy results in a better synergistic effect than monotherapy. From the above studies, it could be concluded about the incompetence of using Photofrin alone for cancer treatment, which further underlines the importance of using a targeted nanocomposite formulation or by combining Photofrin-based PDT with other cancer therapeutic agents for effective cancer treatment.

#### 3.1.2. 5-Aminolevulinic Acid (5-ALA)

5-Aminolevulinic acid (5-ALA) is a naturally occurring amino acid, and biosynthesized ALA can be found in mammals from the Shemin pathway that occurs in mitochondria. The ALA is a precursor of porphyrin, heme, and bile pigments, and it is metabolized into protoporphyrin IX (PpIX) during the course of heme synthesis. PpIX preferentially accumulates in tumor cells, resulting in red fluorescence following irradiation with UV-Vis light and the formation of singlet oxygen, for ALA-induced photodynamic diagnosis and therapy [42]. Babic and co-workers used 5-ALA modified squalene (SQ) in the detection and treatment of tumors using prostate cancer cells PC3 and human glioblastoma cells U87MG [43]. As a natural precursor of cholesterol for targeting tumors, the 5-ALA modified SQ nanocomposite showed promising anti-cancer effects for both cancer cell lines compared to using pristine 5-ALA. Ma and co-workers provided a new strategy for skin cancer treatment by loading 5-ALA within hollow mesoporous silica nanoparticles (HMSNPs) to kill B16F10 skin cancer cells [44]. By using folic acid (FA) as a targeting ligand, 5-ALA loaded nanocomposite shows excellent cytotoxicity towards cancer cells for PDT in vitro. Choi et al. used liposomes to encapsulate 5-ALA for in vitro PDT of human cholangiocarcinoma cells (HuCC-T1) [45]. The 5-ALA encapsulated liposomes showed enhanced phototoxicity due to better intracellular uptake by cancer cells than free 5-ALA. Wang and co-workers used 5-ALA loaded mesoporous organosilica coated Prussian blue nanoparticles (PS@PMOs) for glioma treatment against a malignant glioma cell line (U87MG) [46]. They found effective phototoxicity of 5-ALA after loading in the nanoparticles to show photodynamic therapeutic effects. In a different study, Wang et al. used poly(lactic-co-glycolic acid) (PLGA) nanoparticles (NPs) to deliver 5-ALA for PDT in skin cancer treatment [47]. From in vivo studies, they found that PS-loaded NPs showed more cytotoxicity toward the squamous cell carcinoma (SCC) cell line. To improve PDT, Chen et al. used nanoethosomes (ES) to load 5-ALA, followed by conjugating with hyaluronic acid (HA) as a targeting ligand for synergistic transdermal delivery [48]. The hypertrophic scar fibroblasts (HSFs) cell line was used for in vivo and in vitro studies. Embedding 5-ALA within ES and conjugating the nanocomposite with HA provides effective targeted transdermal delivery to significantly elevating PDT efficacy.

**Figure 3:**
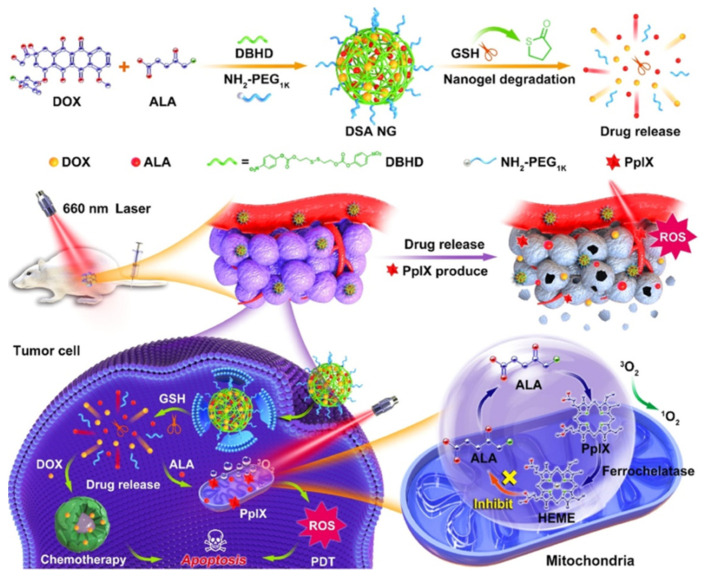
A glutathione-responsive multifunctional nanogel loaded with 5-aminolevulinic acid (5-ALA) photosensitizer and doxorubicin (DOX) for chemo/photodynamic combination cancer therapy. Reproduced with permission from [49]. Copyright 2010. American Chemical Society.

In recent years, combination cancer therapy has received significant attention. For this reason, Kang and co-workers developed a combination cancer therapy approach (chemotherapy plus PDT) using glutathione-responsive multifunctional nanogel for cancer treatment (Figure 3) [49]. With 5-ALA for PDT and doxorubicin (DOX) as a chemo drug, the “Trojan Horse” nanogel shows effective response against the 4T1 cancer cell line. For glutathione depletion, Li et al. used dual-targeted 5-ALA for enhanced PDT [50]. By using ALA methyl ester (OMe), which can be metabolized to PpIX inside the cells, the combination of dual targeting with PDT provides enhanced cancer therapeutic efficacy.

#### 3.1.3. Chlorin e6 (Ce6)

Chlorin e6 (Ce6) was considered one of the most efficient first-generation PSs due to its low dark toxicity and excellent anticancer properties. In 1993, Goff and co-workers introduced immune-conjugate by binding a monoclonal antibody (OC125) with Ce6 for anticancer treatment, which shows excellent in vivo phototoxicity in an animal model created with antigen CA 125-overexpressing ovarian cancer cells [51]. After multiple low-dose treatments, the number of cancer cells was significantly reduced with no treatment-related deaths. In another research, Feng et al. employed a new approach by using diselenide (Se-Se) bonds to link hyaluronic acid (HA) and Ce6, followed by self-assembly into HA-Se-Se-Ce6 micelles [52]. The HA was used as hydrophilic shell to target cluster of differentiation 44 (CD44) receptors overexpressed on 4T1 cancer cells, while PS could be released from the Ce6-containing core after dissembling of micelles in an intracellular redox environment. The targeted delivery of Ce6 to 4T1 tumors was proved in an orthotopic mammary fat pad tumor model for enhanced PDT of breast cancer. Liu and co-workers combined chemotherapy, PDT, and PTT for cancer treatment using reduced graphene oxide (rGO) as a drug carrier, doxorubicin (DOX) as a chemotherapy drug, and Ce6 as PS [53]. In vitro studies showed that Ce6-based combination treatment results in better results than using free Ce6 alone. Xu et al. used polyethylene glycol (PEG)-Ce6 chelated gadolinium ion nanoparticles (PEG-Ce6-Gd NPs) for cancer diagnosis and treatment, which were synthesized via a self-assembly approach [54]. In pre-clinical studies, PEG-Ce6-Gd NPs were verified as promising non-toxic nano-agents for PDT and for contrast-enhanced MRI diagnosis. The synthesized NPs were able to significantly increase their phototoxicity under laser irradiation, inducing the death of cancer cells. Using C6 cell line in vitro and in vivo, the nanotheranostic agent PEG-Ce6-Gd NPs could facilitate diagnosis and PDT treatment of glioma xenografts in mice. To enhance PDT, Liu and his research team doped graphene oxide nanosheet with MnO_2_ for chemo-photodynamic combinatorial therapy after loading cisplatin and Ce6 and surface conjugation with HA targeting ligand [55]. Since a tumor shows extreme hypoxia pathologically, MnO_2_ doping can catalyze the decomposition of H_2_O_2_ into oxygen to alleviate tumor hypoxia and fortify ROS generation from Ce6 after 635 nm laser irradiation.

Hu et al. conjugated IR820 with d-α-tocopheryl polyethylene glycol 1000 succinate (TPGS) for encapsulation of Ce6 in TPGS-IR820/Ce6 micelles [56]. The TPGS-IR820/Ce6 micelles are endowed with multiple theranostic properties, including fluorescence imaging, PTT, and PDT. The stable micelles have a high singlet oxygen production capability as well as remarkable photothermal conversion efficiency. Following effective cellular internalization, a single NIR laser irradiation of the micelles leads to remarkable anticancer activity in vitro and in vivo. As it is important not to damage surrounding healthy tissues during PDT in cancer treatment, Kaščáková and co-workers employed targeted PDT through vascular and cellular targeting, with the overexpressed neuropeptide somatostatin receptor (sst_2_) on tumor cells and neovascular-endothelial cells [57]. They synthesized two Ce6 derivatives, Ce6-K_3_-[Tyr3]-octreotate and Ce6-[Tyr3]-octreotate-K_3_-[Tyr3]-octreotate for PDT treatment, using human erythroleukemic K562 cells. The first derivative was demonstrated to show better anticancer properties than the second one due to the difference in hydrophobicity. Lee and co-workers used Er-doped NaYF_4_:Yb,Er,Nd@NaYF_4_:Nd up conversion nanoparticles (UCNPs), emitting dual red and green signals upon NIR laser irradiation, for enhanced PDT against B16BL6 melanoma cells [58]. By incorporating dual PSs (Ce6 and Rose Bengal), both PSs received red and green emissions from Er-doped UCNPs and generated abundant cytotoxic ROS to destroy cancer cells using an 808 nm wavelength excitation light source. Most importantly, their study revealed the use of dual PS is preferred over single PS, with higher ROS generation rates, to improve the efficacy of PDT.

#### 3.1.4. Rose Bengal (RB)

Rose Bengal (RB), a xanthine dye-based PS with incorporated halogen atoms chlorine and iodine to increase anticancer properties, is widely used as a fluorescein in biological applications. In particular, the heavy atoms in the xanthene rings enhances spin-orbit coupling and promotes intersystem crossing in an excited state of the PS, leading to increased triplet state population and ROS generation. This PS is mainly excited at longer wavelengths (>640 nm), which allows deep light penetration to cause phototoxicity. Furthermore, the short tissue accumulation durations of RB, usually less than two weeks, allows early patient release from the dark. However, like most second generation PSs that are hydrophobic with poor water solubility, RB is prone to aggregation under physiological settings, lowering the quantum yields of ROS generation significantly. Multiple cell death mechanism, including apoptosis, autophagy, and necrosis, are efficiently triggered by this PS, all of which occur independently of one another. Because of its photo-bleaching feature, RB is generally accepted as a PS with few adverse effects [59,60].

Sun et al. developed a cancer treatment modality by combining radiation therapy (RT) with X-ray-induced photodynamic therapy (X-PDT) for clinical deep penetrating cancer therapy. They used RB-doped silica for PDT, together with arginylglycylaspartic acid (RGD) peptide, as a targeting agent for treating U87 malignant glioma cell line in vitro and in animal studies [61]. The results show that low dose X-ray irradiation, as well as using RB as a PS and RGD as a targeting ligand, can overcome the limitation of RT and PDT in cancer treatment. Gianotti and co-workers developed a modified nanosystem with mesoporous silica (MSNs) as a nanocarrier for RB for treating skin cancer [62]. After green light irradiation, RB loaded within MSNs function as a PDT agent and generated ROS to reduce the proliferation of human melanoma SK-MEL-28 cells. Wang and co-workers developed novel nanoplatforms for phototherapy of oral cancer with RB as a photodynamic agent and gold nanorods (GNRs) as a photothermal agent [63]. Green laser light was used to activate RB and red laser light for GNRs during in vitro study with Cal-27 cells. In vivo study with hamster cheek pouches, an animal model found RB-GNRs with combined PDT/PTT, can provide enhanced anti-cancer efficacy against oral cancer.

**Figure 4:**
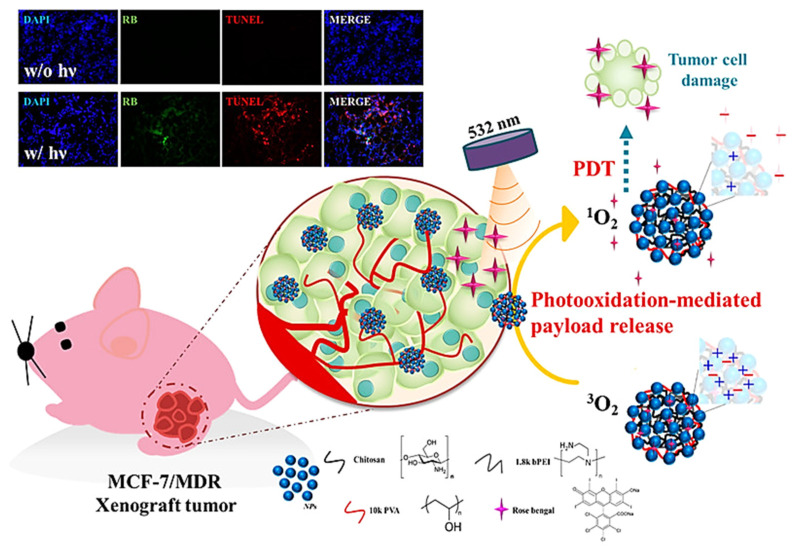
Encapsulation of Rose Bengal (RB) photosensitizer within chitosan/poly (vinyl alcohol) (PVA)/branched polyethylenimine (bPEI)/hydrophobic magnetic nanoparticles nanocomposites through electrostatic interaction provide a ROS-responsive photo-oxidation-responsive nanoplatform for controlled paclitaxel release and photodynamic therapy. Reproduced with permission from [64]. Copyright 2018. American Chemical Society.

For the controlled release of RB by light irradiation, Yeh et al. developed a nanoplatform by encapsulating RB within nanocomposites of chitosan (CTS)/poly (vinyl alcohol) (PVA)/branched polyethylenimine (bPEI)/hydrophobic magnetic nanoparticle through electrostatic interaction [64]. Release of the PS and a chemo drug (paclitaxel) from the nanoclusters could be achieved simultaneously after laser light irradiation through a ROS-responsive photo-oxidation mechanism sensitized by RB (Figure 4). They demonstrated highly effective PDT or PDT-combined therapy against MCF-7 breast cancer cells, SKOV-3 ovarian cancer cells, and Tramp-C1 prostate cancer cell lines in vitro, as well as enhanced PDT efficacy against multidrug-resistant MCF-7/MDR tumors in xenograft animal model. Jain et al. synthesized GAG@mSiO_2_@RB nanocomposite by loading RB in mesoporous silica (mSiO_2_)-coated magnetic luminescent Gd_2.98_Ce_0.02_Al_5_O_12_ nanoparticles (GAG) for X-rays PDT (X-PDT) [65]. The GAG@mSiO_2_@RB nanocomposite produced four times more singlet oxygen when exposed to low-energy X-rays and showed excellent PDT effect upon irradiation with blue light laser against MDA-MB-231 cells, as compared to RB alone. Combining X-PDT with imaging property, the GAG@mSiO_2_@RB nanocomposite is suitable for the theranostic application of deep tumors without the limitation of restricted light penetration depth.

Liu and co-workers used self-assembled RB-loaded peptido-nanomicelles (RBNs) for cancer treatment [66]. The nanocomposite exhibited excellent synergistic effect towards nasopharyngeal carcinoma (NPC) cells from combination sonodynamic therapy (SDT), PDT and chemotherapy (CT). In vitro and in vivo studies in nude mice verified RBNs deliver effective payloads for NPC-targeted cell killing. The combination therapy demonstrated an overwhelming tumor inhibition and lesion elimination capability due to the synergy-enhanced approach. Overall, RBN-mediated combination SDT/PDT/CT provides a promising approach for clinical NPC treatments in a non-invasive, efficacious, and precise manner. Additional efforts by Zhang et al. used carboxymethyl chitosan (CMCS)-RB-DOX nanoparticles for co-delivery of RB and DOX [67]. The combination of PDT with chemotherapy leads to better inhibition effects on Cal-27 oral cancer cells with a sustained intracellular release of RB and DOX. Aside from those reports, other studies also support the use of RB-loaded nanocomposites in combination cancer therapy, which can synergistically improve cytotoxicity against cancer cells and exhibit better cancer treatment outcomes than using PDT alone [68,69,70].

### 3.2. NIR Photosensitizers

The near infrared-based PSs are employed more frequently due to their higher ROS production when induced by longer wavelengths light sources that are endowed with deeper tumor penetration depth. Due to the lack of a long-wavelength absorption band, lower wavelength PSs are limited by their inability to penetrate tissue and elicit abundant photo-induced damage to tumor tissues [71,72]. Thus, near-infrared (NIR)-activated PDT technology has recently been recognized as a promising method for improving cancer treatment [73]. Among the NIR dyes, ICG is the earliest PS with U.S FDA approval status for both PDT and PTT. However, ICG has several limitations that could be resolved with newly developed NIR PSs (IR780, IR806, and IR820) for cancer treatment.

#### 3.2.1. Indocyanine Green (ICG)

**Figure 5:**
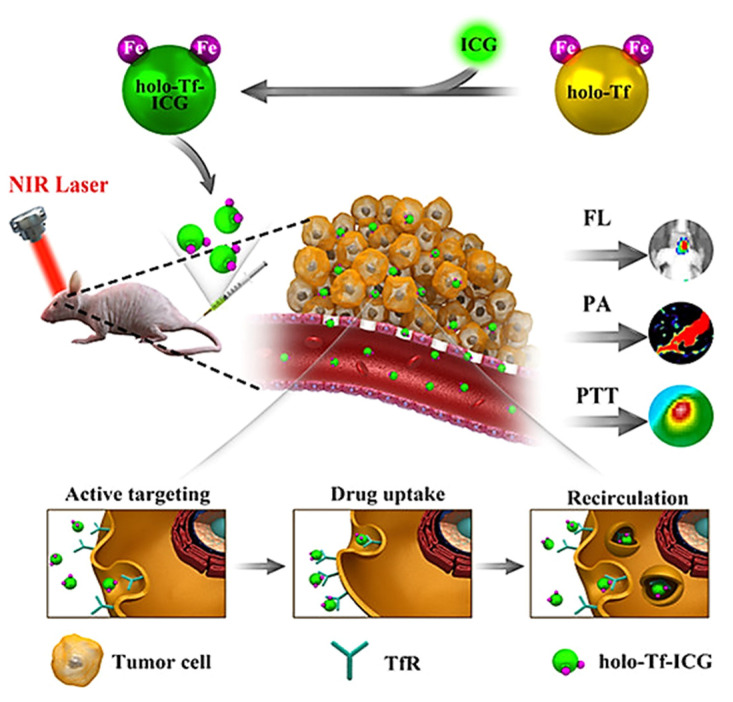
Conjugation of ICG to holo-transferrin (holo-Tf), followed by self-assembly into holo-Tf-ICG nanoparticles for tumor-targeted photoacoustic (PA) and fluorescence (FL) imaging-guided photothermal therapy (PTT) of glioma. Reproduced with permission from [76]. Copyright 2017. American Chemical Society.

Indocyanine green (ICG) is considered an active dye that finds many applications in cancer theranostics by functioning as a photothermal agent (PA), a photosensitizer (PS), or a fluorescence imaging probe. During photothermal treatment of cancer, ICG could convert the optical energy into thermal energy in the presence of NIR light and effectively destroy the tumor. Additionally, ICG also generates ROS to further integrate with their anti-tumor properties, from which dual phototherapy (PTT/PDT) works better than single phototherapy (PTT or PDT) [74]. Sheng et al. used human serum albumin (HAS) and ICG to prepare HSA-ICG NPs by intermolecular disulfide bonds [75]. This nanocomposite shows excellent accumulation and long-term retention in 4T1 tumor-bearing mice from ICG-based NIR fluorescence and photoacoustic dual-modal imaging in vivo, providing image-guided cancer phototherapy with 808 nm laser light to exhibit PDT/PTT synergistic effects. Zhu et al. synthesized a nanocomposite by binding ICG with tumor-targeting ligand holo-transferrin (holo-Tf) through hydrophobic interaction and hydrogen bonds [76]. The holo-Tf-ICG assembly was used for PTT as well as dual-mode (fluorescence and photoacoustic) imaging of U87 orthotopic glioma in nude mice, providing excellent diagnostics and tumor ablation abilities (Figure 5).

Sherien and co-workers carried out a comparative study about using ICG-entrapped polymeric nanoparticles (ICG-ormosil) and free ICG for PDT treatment against adenocarcinoma cells (MCF-7) and hepatocellular carcinoma cells (HepG2) [77]. Both forms of PS (ICG-ormosil and free ICG) showed similar cytotoxic and phototoxic impacts on MCF-7 and HepG2 cell lines. Nonetheless, the entrapment of ICG in polymeric nanoparticles was found to enhance its water stability over free ICG with improved photodynamic activity. Following the same line, Tamai et al. used ICG-loaded super carbonate apatite (sCA) for PDT treatment against HT29 cancer cells [78]. The sCA-ICG outperforms free ICG in cancer treatment in vitro and in vivo. Other than a good carrier for ICG for PDT, sCA was also found to help the quick removal of ICG from other organs, making this nanocomposite a novel tool for cancer research.

As the high concentration of glutathione (GSH) inside tumor cells can consume generated ROS during PDT and lead to insufficient therapeutic effect. Realizing this limitation, Hu and co-workers combined the use of a GSH-depletion agent, phenethyl isothiocyanate (PEITC), with ICG-encapsulated hydroxyethyl starch-oleic acid (HES-OA) NPs to increase the impact of PDT [79]. The HES-OA NPs exhibited excellent stability, promoting cellular uptake and enhancing tumor accumulation, and generated effective singlet oxygen with laser light exposure. Using the ICG-loaded NPs together with PEITC was shown to result in better cancer cell killing both in vitro and in vivo by enhancing the photodynamic effect. In a different study, Hu et al. used ICG-loaded polydopamine-reduced graphene oxide nanocomposites (ICG-PDA-rGO) as a theranostic agent for amplifying photoacoustic (PA) imaging and PTT [80]. The PDA layer coated on rGO surface was shown to promote loading of ICG molecules, quench ICG fluorescence, and enhance optical absorption at 780 nm. Together, the ICG-PDA-rGO nanocomposite showed a stronger PTT effect and better PA contrast than pure GO and PDA-rGO, as well as suppressing 4T1 tumor completely in vivo. Our group developed a novel carrier for ICG in dual-targeted (magnetic and HA ligand) PTT/PDT [81]. From in vitro and in vivo experiments, magnetic liposome (MPLs) encapsulating ICG was coated with hyaluronic acid-polyethylene glycol (HA-PEG) for targeting the U87MG cell line. Intravenous administration of HA-PEG-MPLs prevents tumor growth after successive short-term NIR laser irradiation in vivo.

With encouraging results shown from the combination of chemotherapy (CT) and PTT for improving cancer therapeutic efficacy, a dual-responsive, folic acid (FA)-decorated polymeric micelles (FA Co-PMs) was developed by Zhang et al. for targeted NIR imaging and combination PTT/CT by encapsulating DOX and ICG [82]. The nanocomposite showed triggered DOX release in response to intracellular acidic pH/reduction environments as well as good photothermal response to NIR laser. With significant targeting of BEL-7404 cells and laser-induced hyperthermia, FA Co-PMs synergistically induced death of BEL-7404 cells by apoptosis in vitro and suppressed tumor growth in BEL-7404 xenograft in vivo. A tumor-targeted mesoporous silica nanoparticle (MSN) was designed by Lei and co-workers to load ICG and DOX for NIR-induced photothermal drug release in combined CT/PTT [83]. The nanocomposite was smartly modified with a thermal-cleavable gatekeeper, which can be de-capped by ICG-generated hyperthermia under NIR illumination to trigger DOX release after intracellular uptake for combined cancer therapy. Finally, a similar system for combination therapy was designed based upon poly(γ-glutamic acid)-g-poly(lactic-co-glycolic acid) (γ-PGA-g-PLGA) nanoparticles for loading DOX and ICG [84]. After coating with cholesterol-PEG for alleviating multidrug resistance (MDR), the nanocomposite could effectively treat human MDR breast cancer with combined CT/PTT.

#### 3.2.2. IR780 Iodide

The lipophilic dye IR780 iodide accumulates specifically in breast cancer cells as well as human lung cancer cells, with peak emission at 780 nm to be easily detected from NIR fluorescence imaging. The use of IR780 for prostate cancer imaging has been intensively researched to increase its therapeutic efficacy [85]. With considerable optical absorption and emission in the NIR region, IR780 has attracted the attention of researchers working in the fields of cancer treatments and imaging modalities. When IR780 is exposed to NIR light, it generates a significant amount of ROS and shows good photothermal conversion, making it an active agent for use in cancer photodynamic and photothermal treatment [27]. Several deficiencies shown by ICG, including poor stability, concentration-dependent aggregation, short circulation half-life, and off-target effects, prompts the use of different NIR-based dyes as improved PSs [86]. Among them, IR780, a lipophilic cationic heptamethine dye, presents a relatively new NIR dye showing more stability than ICG as well as higher fluorescence intensity than ICG [87,88,89]. When IR780 iodide is exposed to light at 808 nm wavelength, it produces singlet oxygen for PDT. On the other hand, because of the heat generated by laser irradiation, IR780 may also be employed as a photothermal agent for PTT. To overcome the weak water solubility and low tumor-targeting effectiveness of IR780 iodide, Wang et al. conjugated IR780 to transferrin (Tf), followed by self-assembly into IR780-loaded Tf nanoparticles (NPs) for targeted imaging and PDT/PTT, with photothermal response as well as singlet oxygen generation under 808 nm laser irradiation [90]. In vitro and in vivo studies demonstrated that loading IR780 in Tf NPs could destroy cancer cells more effectively. Xing and co-workers developed a photo-responsive nanocluster (NC) system, by the combination of polydopamine (PDA) with TPGS micelles for loading IR780 and DOX, for combined chemo-phototherapy against breast cancer cells [91]. Their study confirms the use of DOX as a chemo drug to amplify the efficiency of PTT/PDT in suppressing multidrug resistance (MDR) breast cancer.

As stated earlier, the efficacy of PDT is largely limited by oxygen deficiency in the hypoxic tumor microenvironment, even with IR780. To solve this problem, Yang and co-workers fabricated a NIR-responsive nanocomposite for co-delivery of oxygen and DOX, by loading DOX in an oxygenated amphiphile (F-IR780-PEG) to form F/DOX nanoparticles [92]. The intrinsic fluorescent features of IR780 make F/DOX nanoparticles suitable for the imaging-guided treatment of hypoxic tumors with synergistic CT/PDT. Yue et al. developed a nanoplatform based on thermosensitive liposomes for loading IR780 as well as Lonidamine as an anti-cancer drug [93]. By releasing ROS for PDT in mitochondria using an 808 nm light source, the IR-780-entrapped liposomal formulation increased anti-cancer efficacy for lung cancer treatment with effects from combination cancer therapy. Yuan et al. loaded IR-780 in PEG-C13 micelle as a targeted photothermal and imaging agent for cancer therapy [94]. The self-assembled micelle showed good phototherapy efficacy both in vitro and in vivo.

**Figure 6:**
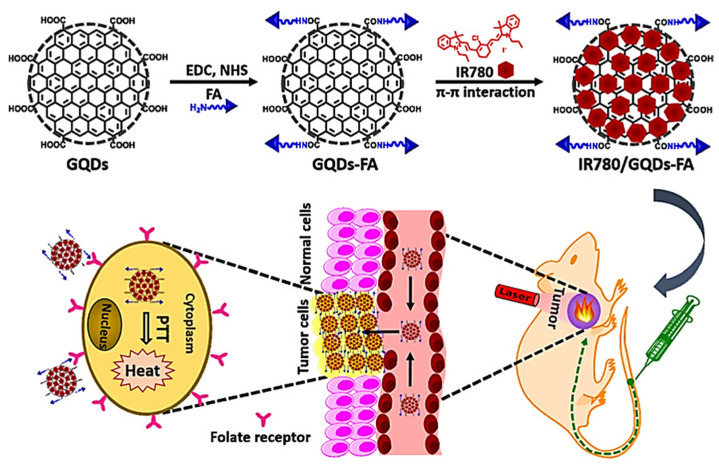
Loading IR780 iodide on folic acid (FA)-functionalized graphene quantum dots (GQDs) for targeted photothermal therapy (PTT). Reproduced with permission from [95]. Copyright 2017. American Chemical Society.

As IR780 exhibits insoluble properties in all clinically used solvents, Li and co-workers used folic acid-conjugated graphene quantum dots (GQDs-FA) for loading IR780 (Figure 6) [95]. With an improved loading capacity of IR780 through strong π−π stacking, over 2400-fold higher aqueous concentration of IR780 could be achieved using IR780/GQDs-FA. Loading IR-780 by non-covalent interaction further enhances its photostability, which upon laser treatment, produces efficient hyperthermia to kill HeLa cancer cells. Wang et al. employed IR780 as a PDT agent for combination with immunotherapy using anti-PD-L1 peptide [96]. Their innovative design provides an IR780-loaded nanocomposite for effectively eradicating metastatic and invasive tumors as well. Under laser light irradiation, IR780 destroys tumor cells by creating ROS, while PDT-induced immunogenic cell death exposes tumor-associated antigens and damage-associated molecular patterns, allowing dendritic cells to mature and cytotoxic T lymphocytes to activate cytotoxic T lymphocytes for immunotherapy.

#### 3.2.3. IR806

The IR806 is a negatively charged hydrophilic PS with a sulfonate functional group that enhances its hydrophilicity, which is missing in IR780. This difference also prevents agglomeration and less toxicity associated with IR806 in comparison to hydrophobic IR780 [97,98]. Chang et al. synthesized a nanocomposite based on IR806 for dual (fluorescence and magnetic resonance) image-guided PTT [24]. After functionalizing IR780-iron oxide complex with mPEG-PCL-G2.0PAMAM-Cit for tumor targeting, the enhanced photothermal conversion efficiency originated from both the PS and iron oxide nanoparticles provide effective PTT with low-power (0.25 W/cm^2^) 808 nm lasers. Similarly, for magnetic targeting as well as image-guided PTT/PDT using 808 nm laser, Deng et al. developed a nanocomposite with IR806 and MnFe_2_O_4_ (MFO-IR) for magnetic-targeted/MRI-guided synergistic PTT/PDT [99]. After derivatization with carboxyl groups, IR806 was conjugated to MnFe_2_O_4_ nanoparticles, showing multi-functionality as PS carriers, targeting ligands, MRI contrast agents, and photothermal agents. In vitro experiments show NIR light-responsive ROS generation for cell apoptosis and hyperthermia with HeLa cells. After guidance with an external magnetic field and followed by 808 nm laser irradiation, the nanocomposite could completely eradicate subcutaneous tumors in vivo.

Using 793 nm NIR laser for improving tissue penetration and reducing overheating by water absorption, Lin et al. synthesized IR806-loaded neodymium up-conversion nanoparticles (UCNPs) for heat generation and luminescence imaging [100]. From in vitro and in vivo studies performed against MDA-MB-231 cancer cells using polyethylene glycol-folic acid (PEG-FA) as a tumor targeting agent, potential theranostic cancer treatment was successfully demonstrated from this IR806-loaded nanocomposite. Jogdand and co-workers entrapped IR806 in chitosan-coated niosome (NioIR-C) to enhance the photothermal conversion efficacy of IR806 by chitosan [101]. The mucoadhesive nature of NioIR-C and its intracellular uptake by cancer cells enhanced the PTT therapeutic effect against MCF-7 and MDA-MB-231 breast cancer cells. Using the additive nature of PTT/PDT in the central nervous system, Wang et al. used self-assembled Fe_3_O_4_-IR806 as a nanocomposite for treating malignant glioma [102]. By complexing with IR806, the nanoplatform shows a 3.5-fold increase of photothermal conversion efficiency over that of Fe_3_O_4_ nanoparticles for PTT, which also generates ROS for PDT with NIR light irradiation. The nanocomposite suppressed the growth of glioma cells by photothermal ablation and generated ROS cytotoxicity in vitro, in addition to inhibiting tumor growth in vivo with NIR light induction. A limiting factor in PDT is the short-life and restricted diffusion distance of ROS generated by a PS. Therefore, Yu et al. used IR806 to develop a mitochondria-targeted NIR-responsive TiO_2_-coated UCNPs nanocomposite for PDT [103]. By localizing into mitochondria through intracellular trafficking followed by NIR laser irradiation, the nanocomposite could produce ROS in mitochondria and induce the domino effect on ROS burst, which leads to mitochondria collapse and cell apoptosis with overproduced ROS accumulated in mitochondria. This enhanced PDT strategy was proved to be effective for the complete removal of the tumor in vivo. Using gel matrix as a scaffold for the administration of PS has rarely been reported. For this purpose, Asadian-Birjand and co-workers conjugate IR806 into a nanogel of dendritic polyglycerol (dPG) and oligo ethylene glycol (OEG) [104]. Improved phototoxicity of the dye-nanogel nanocomposite against a human carcinoma cell line after NIR laser irradiation in vitro implicates its potential use as a photothermal and photodynamic agent in cancer therapy.

#### 3.2.4. IR820

The scientific community has shown extensive interest in NIR-based fluorophores due to their combined molecular imaging and hyperthermia properties. The cyanine dye IR820 exhibits optical and thermal properties similar to those of ICG, albeit conferred with excellent in vitro and in vivo stability over ICG. Although IR820 shows a lower quantum yield as compared to ICG in fluorescent emission study; however, it shows less dependence of the emission peak location on concentration. Under all temperature and light conditions, IR820 provides a half-life nearly twice that of ICG in aqueous solutions. After 3-min laser irradiation, IR820 produced slightly lower peak temperatures (less than 10%) than ICG in hyperthermia applications [105]. When comparing the heat-generating capacity and imaging properties between ICG and IR820, IR820 showed enhanced stability with nearly doubled degradation half-time and prolonged image collection time, in addition to similar heat generation property with ICG [106]. The negative charge of IR820 can provide stable static interaction with amine groups, although its absorption in the NIR region was weakened [107]. It was shown that IR820 might act both as a PS and a photothermal agent (PA), transferring energy to ^3^O_2_ to produce ^1^O_2_ and generating heat by laser irradiation at 808 nm [108].

**Figure 7:**
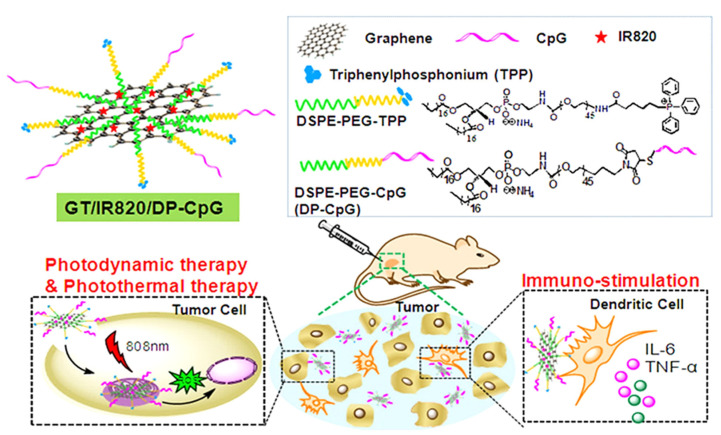
The “triple-punch” anticancer strategy mediated by NIR photosensitizer IR820/CpG oligonucleotides and mitochondria-targeted TPP-modified nanographene for photodynamic/photothermal/immunotherapy. Reproduced with permission from [112]. Copyright 2018. American Chemical Society.

In an interesting work by Xia and co-authors, IR820 was conjugated to porous silicon nanoparticles (PSiNPs) for the controlled release of DOX in cancer therapy [109]. A high drug release percentage (98%) of DOX was noted using NIR-triggered DOX release in acidic endosomal environments. The combination of IR820 and DOX enhanced the chemo-photothermal combination therapy towards HeLa cancer cells in vitro. Consider tumor imaging only, Zhou et al. conjugated IR820 to chitosan quaternary ammonium salt capped ferroferric oxide (CSQ-Fe) nanoparticles. The IR820-CSQ-Fe nanocomposite was used for multimodal MRI, NIR fluorescence imaging as well as multispectral optoacoustic tomography of MDA-MB-231 cancer cells [110]. For solving the poor solubility of paclitaxel (PTX) and short life-time of IR820, an “all-in-one” approach for dual photo-chemo therapy/imaging was suggested by Zhang et al., who prepared IR820-PTX nanocomposite with very high drug content (96% IR820 and PTX) [111]. This enzyme and pH-sensitive conjugates show good stability in the blood stream for NIR fluorescence image-guided CT/PTT. A “triple-punch” strategy of cancer PTT, PDT, and immunotherapy was used by Wu et al., who designed a mitochondria-targeted and NIR-activated multifunctional graphene nanocomposite after modification with triphenylphosphonium (Figure 7) [112]. By generating abundant ROS and photothermal heat for cell apoptosis, this photo-active nanocomposite is confirmed for PTT/PDT in vitro. With the conjugation of lipophilic DP-CpG cytosine-guanosine oligonucleotides to graphene surface by hydrophobic interaction for promoting secretion of pro-inflammatory cytokines and improves tumor immunogenicity, the GT/IR820/DP-CpG is also effective for immunotherapy. From photo-immunotherapy in vivo, an 88% tumor inhibition rate was obtained, implicating PTT/PDT effect from IR820 could be combined with DP-CpG immunostimulation for multimodal cancer therapy.

Zaharie-Butucel et al. developed hybrid nanomaterials by using chitosan-reduced graphene oxide (chit-rGO) as a carrier for delivery of IR820 and DOX in vitro against murine colon carcinoma cells (C26) [113]. In their study, the anticancer activity against C26 cancer cells could be improved by the combination of the photodynamic effect of IR820, the photothermal effect of IR820 and chit-rGO, as well as the chemotherapeutic effect of DOX. Using microneedle (MN) patches to deliver IR820 and cisplatin, Fu and co-workers fabricated a nanodevice for breast cancer therapy by delivery of the PDT and chemotherapy agents from the drug-carrying needle tips, with inserted MN in the skin for local cancer treatment [114]. The developed MN patch was shown to be with low toxicity for controllable synergistic chemo-photodynamic therapy efficacy. Combining PTT with an indoleamine 2,3-dioxygenase (IDO) inhibitor is an attractive approach for immunotherapy. For this purpose, Zhang and co-workers conjugated IR820 with the IDO inhibitor 1-methyl-tryptophan (1MT) to induce immunogenic cell death (ICD), followed by self-assembly into IR820-1MT nanoparticles [115]. This nanocomposite not only showed 90% loading of both therapeutic agents but also solved the poor water solubility of 1MT and the short lifetime of IR820. After triggering by NIR laser, IR820-1MT nanoparticles showed excellent photothermal-assisted immunotherapy against tumor recurrence and metastasis. The heme oxygenase-1 (HO-1) is an antioxidant protein, which is overexpressed in response to the stress experienced by cancer cells during anticancer therapy for regulating cell apoptosis. It is conceivable that reducing the anti-oxidative ability of cancer cells by inhibition of HO-1 activity will make cancer cells more vulnerable to damage caused by photothermal heating during PTT. Following this reasoning, Noh et al. developed a multifunctional nanocomposite by conjugating amphiphilic polymers with zinc protoporphyrin (ZnPP) for inhibition of HO-1, together with IR820 for PTT [116]. The nanocomposite showed enhanced oxidative–photothermal combination anticancer therapy upon NIR laser irradiation by elevating cytotoxicity against cancer cells. An in vivo study with a mouse xenograft model revealed that dual image-guided enhanced PTT could lead to thermal ablation of the tumor without side-effects and tumor recurrence.

A summary of PS/wavelength, the delivery and functionalization agents in the nanocomposites, the cancer cell lines used for the study, as well as the types of study are provided in Table 1.

## 4. Conclusions and Outlook

In this review, the most important and commonly used PSs for delivery by different nanocomposites are discussed in the context of cancer theranostics. While perfect PSs with responsiveness to deep tissue penetration light and high solubility in physiological buffers are still elusive, advances in nanomaterial design by complexing PSs with smartly designed nanocomposites have provided encouraging results in combination cancer therapy based on PS-mediated PTT and/or PDT. Although the second generation PSs has limitations like their first-generation counterparts, their numerous usages in a clinical setting have proven their multi-functionality and efficacy in phototherapy-based cancer treatments guided by different imaging modalities. Among all PSs, the long-wavelength PSs (IR806 and IR820) should provide a better solution to the issues faced during phototherapy if their half-lives could be further extended. Contemporaneous research is still ongoing to develop better PSs that may enhance the photodynamic effect, and more research is warranted for new PSs that could be used for dual PDT/PTT with high efficiency. This could be complemented through the unique design of a nanocomposite that can incorporate PSs with moieties alleviating hypoxia and inhibiting thermal stress protein activity in a single nanoplatform for enhanced PTT/PDT. A better treatment outcome in a clinical setting may be possible if more PSs could be used for detection during cancer treatment to realize new multitasking nanotheranostic tools. Advances in the development of more specific technologies for delivery of PS and/or light source are also urgently needed to achieve the best clinical approach towards cancer treatment.

## Figures and Tables

**Table 1:** Summary of photosensitizer-containing nanocomposites for cancer therapy.

Photosensitizer /Wavelength	Delivery/Functionalization Agents	Cancer Cells	Types of Study	Reference
**Ultraviolet-Visible (UV-Vis) Photosensitizers (PSs)**				
Porphyrin sodium (Photofrin) /510–630 nm	GC	EMT6, 4T1	In vitro, In vivo	[36]
	Zn	MDA-MB-231	In vitro	[37]
	Carboplatin	HeLa	In vitro	[38]
	Chloroquine	HCT116	In vitro, In vivo	[39]
	PLL-g-PEG	4T1, CT26	In vitro, In vivo	[40]
	Cisplatin, Gemcitabine	H1299	In vitro	[41]
5-aminolevulinic acid (5-ALA) /400–660 nm	SQ	PC3, U87MG	In vitro	[43]
	FA, HMSNPs	B16F10	In vitro	[44]
	Liposomes	HuCC-T1	In vitro	[45]
	PB@PMOs	U87MG	In vitro, In vivo	[46]
	PLGA	SCC	In vivo	[47]
	ES, HA	HSFs	In vitro, In vivo	[48]
	DOX	4T1	In vitro, In vivo	[49]
	OMe	HeLa	In vitro, In vivo	[50]
Chlorin e6 (Ce6) /635–808 nm	HA	4T1	In vitro, In vivo	[52]
	DOX, rGO	U87	In vitro, In vivo	[53]
	PEG, Gd	C6	In vitro, In vivo	[54]
	Cisplatin, HA, MnO_2_	MDA-MB-231	In vitro, In vivo	[55]
	TPGS, IR820	B16	In vitro, In vivo	[56]
	K3-[Tyr3]-octreotat	K562	In vitro, In vivo	[57]
	RB, UCNPs	B16BL6	In vitro	[58]
Rose Bengal (RB) /532–808 nm	Silica, RGD	U87	In vitro, In vivo	[61]
	MSNs	SK-MEL-28	In vitro	[62]
	GNRs	Cal-27	In vitro, In vivo	[63]
	CTS, PVA, bPEI	MCF-7	In vitro, In vivo	[64]
	GAG, mSiO_2_	MDA-MB-231	In vitro	[65]
	RBNs	CNE-2Z	In vitro, In vivo	[66]
	CMCS, DOX	Cal-27	In vitro	[67]
**Near infrared (NIR) Photosensitizers (PSs)**				
Indocyanine green (ICG) /807–808 nm	HSA	4T1	In vitro, In vivo	[75]
	Holo-Tf	U87	In vitro, In vivo	[76]
	Ormosil	MCF-7, HepG2	In vitro	[77]
	sCA	HT29	In vitro, In vivo	[78]
	HES-OA	HepG2	In vitro, In vivo	[79]
	PDA-rGO	4T1	In vitro, In vivo	[80]
	MPLs, HA-PEG	U87MG	In vitro, In vivo	[81]
	FA, DOX, Co-PMs	BEL-7404	In vitro, In vivo	[82]
	DOX, MSN, RGD	4T1	In vitro, In vivo	[83]
	γ-PGA-g-PLGA, DOX	MCF-7, MCF-7/MDR	In vitro, In vivo	[84]
Infrared 780 iodide (IR780) /808 nm	Transferrin	CT26	In vitro, In vivo	[90]
	PDA, DOX	MCF-7	In vitro, In vivo	[91]
	Oxygenated amphiphiles, DOX	MCF-7	In vitro, In vivo	[92]
	Liposomes, Lonidamine	LL/2	In vitro, In vivo	[93]
	Micelles, PEG	CT26	In vitro, In vivo	[94]
	FA, GQD	HeLa	In vitro, In vivo	[95]
	Anti-PD-L1 peptide	B16F10	In vitro, In vivo	[96]
Infrared 806 (IR806) /785–980 nm	Iron oxide, mPEG-PCL-G2-Cit	A549	In vitro, In vivo	[24]
	MnFe_2_O_4_	HeLa	In vitro, In vivo	[99]
	Neodymium UCNPs, PEG-FA	MDA-MB-231	In vitro	[100]
	Niosome, chitosan	MCF-7, MDA-MB-231	In vitro	[101]
	Fe_3_O_4_	U87MG	In vitro, In vivo	[102]
	TiO_2_-UCNPs	MCF-7	In vitro, In vivo	[103]
	dPG, OEG	A2780	In vitro	[104]
Infrared 820 (IR820) /785–808 nm	PSiNPs, DOX	HeLa	In vitro	[109]
	CSQ-Fe	MDA-MB-231	In vitro	[110]
	PTX	MCF-7, HeLa	In vitro, In vivo	[111]
	Graphene, DP-CpG	EMT6	In vitro, In vivo	[112]
	Chit-rGO, DOX	C26	In vitro	[113]
	Microneedles, Cisplatin	4T1	In vitro, In vivo	[114]
	1MT	B16F10	In vitro, In vivo	[115]
	ZnPP-conjugated micelles	A549	In vitro, In vivo	[116]
